# Prediction of hospital outcome in septic shock: a prospective comparison of tissue Doppler and cardiac biomarkers

**DOI:** 10.1186/cc8931

**Published:** 2010-03-24

**Authors:** David J Sturgess, Thomas H Marwick, Chris Joyce, Carly Jenkins, Mark Jones, Paul Masci, David Stewart, Bala Venkatesh

**Affiliations:** 1 School of Medicine, The University of Queensland, Princess Alexandra Hospital, Ipswich Road, Brisbane, 4102, Australia; 2 Department of Intensive Care, The Wesley Hospital, Coronation Drive, Brisbane, 4066, Australia; 3 Department of Echocardiography, Princess Alexandra Hospital, Ipswich Road, Brisbane, 4102, Australia; 4 Department of Intensive Care, Princess Alexandra Hospital, Ipswich Road, Brisbane, 4102, Australia; 5 School of Population Health, The University of Queensland, Princess Alexandra Hospital, Ipswich Road, Brisbane, 4102, Australia

## Abstract

**Introduction:**

Diastolic dysfunction as demonstrated by tissue Doppler imaging (TDI), particularly E/e' (peak early diastolic transmitral/peak early diastolic mitral annular velocity) is common in critical illness. In septic shock, the prognostic value of TDI is undefined. This study sought to evaluate and compare the prognostic significance of TDI and cardiac biomarkers (B-type natriuretic peptide (BNP); N-terminal proBNP (NTproBNP); troponin T (TnT)) in septic shock. The contribution of fluid management and diastolic dysfunction to elevation of BNP was also evaluated.

**Methods:**

Twenty-one consecutive adult patients from a multidisciplinary intensive care unit underwent transthoracic echocardiography and blood collection within 72 hours of developing septic shock.

**Results:**

Mean ± SD APACHE III score was 80.1 ± 23.8. Hospital mortality was 29%. E/e' was significantly higher in hospital non-survivors (15.32 ± 2.74, survivors 9.05 ± 2.75; *P* = 0.0002). Area under ROC curves were E/e' 0.94, TnT 0.86, BNP 0.78 and NTproBNP 0.67. An E/e' threshold of 14.5 offered 100% sensitivity and 83% specificity. Adjustment for APACHE III, cardiac disease, fluid balance and grade of diastolic function, demonstrated E/e' as an independent predictor of hospital mortality (*P* = 0.019). Multiple linear regression incorporating APACHE III, gender, cardiac disease, fluid balance, noradrenaline dose, C reactive protein, ejection fraction and diastolic dysfunction yielded APACHE III (*P* = 0.033), fluid balance (*P* = 0.001) and diastolic dysfunction (*P* = 0.009) as independent predictors of BNP concentration.

**Conclusions:**

E/e' is an independent predictor of hospital survival in septic shock. It offers better discrimination between survivors and non-survivors than cardiac biomarkers. Fluid balance and diastolic dysfunction were independent predictors of BNP concentration in septic shock.

## Introduction

Septic shock in adults refers to a state of acute circulatory failure characterized by persistent arterial hypotension unexplained by other causes [1]. Although this clinical syndrome is heterogeneous with regard to factors such as causal micro-organism, patient predisposition, co-morbidity and response to therapy, a key element and unifying feature is the manifestation of cardiovascular dysfunction. Although the underlying cause of death in septic shock is often multifactorial, refractory hypotension and cardiovascular collapse are frequently observed in the terminal phases of the condition [2]. Whilst impaired systolic function has been identified as the major culprit, the contribution of diastolic dysfunction (and hence ventricular filling) to cardiovascular morbidity and mortality in septic shock is not fully understood. Investigation of left ventricular (LV) diastolic function at the bedside is challenging, but techniques such as echocardiography and biomarkers such as B-type natriuretic peptide (BNP) are increasingly supported by current literature [3-5]. In particular, recent application of non-invasive, bedside technologies, such as tissue Doppler imaging (TDI), offer fresh insight [6].

TDI is an echocardiographic technique that measures myocardial velocities [7], which are low frequency, high-amplitude signals filtered from conventional Doppler imaging [8]. TDI has gained acceptance amongst cardiologists for the evaluation of diastolic function, particularly as a measure of ventricular relaxation and ventricular filling pressure [9]. However, there are scant data regarding its use in critical care. TDI has demonstrated prognostic utility in a range of cardiovascular diseases [10], including following myocardial infarction [11,12], heart failure [13-16], abnormal LV function at dobutamine echocardiography [17], nonvalvular atrial fibrillation [18], hypertension [19], and end-stage renal disease [20].

Previously, we demonstrated that evidence of diastolic dysfunction on TDI is common in critically ill patients [21]. The significance of this was recently highlighted by Ikonomidis and colleagues, who demonstrated that TDI may be prognostically useful in the general ICU population [22]. To date, the prognostic significance of this technique has not been specifically evaluated in septic shock.

Cardiac biomarkers including BNP [23,24], N-terminal proBNP (NTproBNP) [25] and troponin [26] potentially offer prognostic information in the critically ill. To date, no comparison has been made between TDI and cardiac biomarkers (BNP, NTproBNP and troponin) with regard to prediction of hospital outcome in septic shock.

This study sought to evaluate and compare the prognostic significance of TDI variables and cardiac biomarkers in septic shock. An auxiliary aim was to evaluate the potential contribution of LV diastolic dysfunction and fluid management to elevation of plasma BNP concentrations in septic shock.

## Materials and methods

This prospective observational study was approved by the Princess Alexandra Hospital Human Research Ethics Committee (project 2005/213), and the Guardianship and Administration Tribunal of Queensland (project 2006/07) and informed consent was obtained from the patient or legally authorized representative where appropriate.

### Patients

Twenty-one consecutive adult patients with septic shock were recruited from the ICU during an 11-month period (May 2005 to March 2006). Eligible patients were enrolled within 72 hours of admission to the ICU with septic shock or development of septic shock while in the ICU. Septic shock was defined as severe sepsis with persistent hypotension (ie. with a mean arterial pressure (MAP) < 60 mmHg or a reduction in systolic blood pressure (SBP) > 40 mmHg from baseline) despite adequate volume resuscitation in the absence of other causes for hypotension [1].

Exclusion criteria included: age younger than 18 years; presence of moderate to severe valvular heart disease; or patient or legally authorized representative declined participation.

Patient care followed standard practice. Clinical fluid resuscitation and management were undertaken in a fashion consistent with surviving sepsis guidelines [27]. More specifically, fluid challenges were undertaken incrementally while clinical response was observed. Therapeutic variables considered in determining the requirement and response to fluid management included pulse rate, blood pressure (target MAP > 65 mmHg), peripheral perfusion, urine output (target > 0.5 ml/kg/hr), and central venous pressure (CVP). Research measurements were not released to the treating clinician.

### Clinical and outcome data

Clinical data included height, weight, ventilation mode and settings, heart rate, rhythm, arterial blood pressure (SBP; diastolic blood pressure (DBP); MAP) and CVP. Body surface area (BSA) was calculated [28]. ICU fluid balance was recorded for the study day (fluid balance). Vasopressor/inotropic infusion rates and, ICU and hospital length of stay and outcome were recorded. Illness severity was quantified using (Acute Physiology and Chronic Health Evaluation) APACHE III and Sequential Organ Failure Assessment (SOFA) scores. Patients were considered to have a history of cardiac disease if they had prior or current ischemic heart disease (angina or myocardial infarction) or cardiac surgery.

### Echocardiography

Transthoracic echocardiography and Doppler examinations were performed by experienced echocardiographers (coordinated by Jenkins C) using commercially available echocardiographic equipment (Acuson Sequoia, Siemens AG, Munich, Germany and Sonos 7500, Philips Medical Systems, Andover, MA, USA). Measurements were made off-line, using AccessPoint™ 2000 software (Freeland Systems, Westfield, IN, USA). Unless otherwise stated, measurements were made in triplicate at end expiration.

#### Two-dimensional echocardiography

LV end-diastolic volume (LVEDV) and LV end-systolic volume (LVESV) were calculated using the biplane method of disks (modified Simpson's rule) from the apical four-chamber and two-chamber views [29] and indexed to BSA (LVEDVI and LVESVI, respectively). LV ejection fraction (LVEF) was calculated as (LVEDV - LVESV)/LVEDV × 100. Systolic dysfunction was defined as EF below 55%. LV outflow tract diameter (OTD) was recorded as the maximum measurement from triplicate zoomed parasternal long axis view.

#### Doppler echocardiography

Transmitral flow velocities were recorded with pulsed-wave Doppler with the sample volume placed at the mitral valve tips from the apical four-chamber view [30]. Peak passive (E) and active (A) velocities were recorded. E wave deceleration time (DT) was measured. E to A ratio (E/A) was calculated.

Doppler interrogation of LV outflow tract velocity was guided by apical five-chamber view [30]. Heart rate (HR), velocity time integral (VTI) and peak velocity (Vpeak) were measured. Stroke volume was calculated as the product of VTI and cross-sectional area of the LV outflow tract [π.(OTD/2)^2^]. Cardiac output was calculated as the product of stroke volume and HR. Stroke volume and cardiac output measurements were indexed to body surface area (SVI and CI, respectively).

#### Tissue Doppler

Myocardial velocities were obtained using tissue Doppler settings, with the pulsed-wave Doppler sample volume at the septal mitral annulus in the apical four-chamber view. Peak systolic (s'), early diastolic (e') and late diastolic (a') myocardial velocities were measured. E/e' was calculated. When A and/or a' were indistinguishable due to sinus tachycardia, E and/or e' were measured as described by Nagueh and colleagues [31]. In the presence of atrial dysrhythmia, transmitral and tissue Doppler velocities were measured over five consecutive cardiac cycles [18].

As previously described [21], thresholds for abnormal diastolic TDI were accepted as e'less than 9.6 cm/s (myocardial relaxation below the lower 95% confidence limit of normal subjects) [32] or E/e' more than 15 (mean LV end-diastolic pressure > 15 mmHg) [33].

#### Diastolic dysfunction

Guidelines previously published by our group were used to grade LV diastolic function as normal, impaired relaxation, pseudonormal or restrictive [34]. Age-dependent thresholds for deceleration time (< 40 years < 220 ms; 40 to 60 years 140 to 250 ms; > 60 years 140 to 275 ms) were used to determine impaired relaxation (DT above normal limit) and restrictive patterns (DT below normal limit). In order to distinguish between normal and pseudonormal patterns, we incorporated E/e' (normal < 8; pseudonormal > 15). Where E/e' was inconclusive (8 to 15), increased left atrial area (> 20 cm) was used as a marker of raised LV filling pressure (pseudonormal pattern). Patients categorized other than normal were considered to have diastolic dysfunction.

### Biochemical assay

Plasma BNP concentration was measured using a Biosite Triage^® ^immunoassay (Biosite Diagnostics, San Diego, CA, USA), Plasma Troponin T (TnT; Elecsys^® ^Troponin T, 3^rd ^generation immunoassay; Roche Diagnostics Australia Pty Ltd, Castle Hill, NSW, Australia) and NTProBNP concentration (Elecsys^® ^proBNP, Roche Diagnostics Australia Pty Ltd, Castle Hill, NSW, Australia) were run on Roche Elecsys^® ^analyzers (Roche Diagnostics Australia Pty Ltd, Castle Hill, NSW, Australia). Plasma C reactive protein (CRP) concentration was measured using an immunotubidometric assay (UniCel^® ^DxI 800 Access^® ^Immunoassay System, Beckman Coulter Australia Pty. Ltd., Gladesville, NSW, Australia). Laboratory thresholds were used to determine elevation of biomarkers: BNP (normal < 100 ng/L), NTproBNP (0 to 50 years < 450; 50 to 75 years < 900; > 75 years < 1800 ng/L), TnT (< 0.03 μg/L) and CRP (< 5.0 mg/L).

### Blinding

Coded echocardiographic and Doppler recordings were analyzed at least one month after acquisition by a single observer blinded to clinical and biochemical data. Biochemical assay was performed on coded samples by technicians blinded to clinical and echocardiographic data.

### Statistics

Analysis was performed by SPSS, version 14.0 for Windows (SPSS Inc., Chicago, IL, USA). Descriptive measures were used to evaluate the distribution of variables. Differences between groups were assessed using Fisher's exact test for categorical data. Continuous data were assessed using Levene's test for equality of variance before applying Student's *t*-test for independent samples. BNP and NTproBNP concentrations were log-transformed to achieve normality before application of linear regression techniques. Discrimination between hospital survivors and non-survivors was evaluated by receiver operating characteristic (ROC) curve analysis.

Cox proportional hazards regression was used for time to event outcomes (hospital survival) from the time of echocardiography. Adjustment was made for the potential influence of cardiac disease, fluid balance and grade of diastolic dysfunction upon E/e'.

Multiple-linear regression analyses were undertaken to determine contributions to BNP concentration (lnBNP). Potential predictor variables included APACHE III score (first ICU day), gender, [35], cardiac disease [35], intravenous fluid therapy [36], noradrenaline dose [37], CRP [38], LVEF [39], and LV diastolic dysfunction [40]. A backwards elimination procedure was then used to discard predictor variables with *P *< 0.1 in multiple regression models one by one until a final 'best' model was achieved.

In final analyses, a *P*-value less than 0.05 was regarded as significant. Unless stated otherwise, results are reported as mean ± standard deviation (SD) (range).

### Sample size

Subgroup analysis of septic patients from data previously published by our group yielded a mean ± SD E/e' of 11.4 ± 5 (range: 3.59 to 23.15) and hospital mortality of 30% [21]. It was determined that a sample of 20 patients would allow detection of a mean difference in E/e' of 4 or more between survivors and non-survivors (80% power; α = 0.05) [41].

## Results

### Patient characteristics

Twenty-one consecutive septic shock patients were studied (Table 1). Fifteen participants (71%) were studied within 24 hours of developing septic shock. Variables recorded on the study day are presented in Table 2.

**Table 1:** Patient characteristics

Total number of patients	21
Male:Female ratio	13:8
Age, years	65 ± 17 (24-86)
Height, cm	167 ± 7 (156-180)
Weight, kg	80 ± 18 (42-130)
Body surface area, m^2^	1.88 ± 0.25 (1.4-2.5)
APACHE III score (Day 1 ICU)	80.1 ± 23.8 (46-141)
SOFA score (Day 1 ICU)	11 ± 2.8 (6-16)
ICU length of stay, days	12.5 ± 12.3 (1-54)
Hospital length of stay, days	29.6 ± 29.3 (1-125)
ICU mortality, n (%)	4 (19%)
Hospital mortality, n (%)	6 (29%)
28-day mortality, n (%)	6 (29%)
***Source of infection***	
Abdominal, n (%)	8 (38%)
Pulmonary, n (%)	7 (33%)
Neurologic, n (%)	2 (9.5%)
Necrotizing fasciitis, n (%)	2 (9.5%)
Catheter related sepsis, n (%)	1 (5%)
Mediastinitis, n (%)	1 (5%)
***Cardiac disease, n (%)***	*9 (43%)*
Angina, n (%)	3 (14%)
Myocardial infarction, n (%)	6 (28%)
Cardiac surgery, n (%)	5 (24%)

APACHE, Acute Physiology and Chronic Health Evaluation; SOFA, Sequential Organ Failure Assessment.

**Table 2:** Variables measured on study day

Variable	Mean ± SD (Range)
**Day of study**	
APACHE III score	82.9 ± 29.6 (28-141)
SOFA score	11.6 ± 3.6 (5-19)
**Echocardiography**	
LVEDVI, mL/m^2^	65.8 ± 22.4 (31.9-121.8)
LVESVI, mL/m^2^	37.5 ± 18.5 (13.9-83.2)
SVI, mL/m^2^	26.6 ± 14.5 (8.3-67.9)
EF, %	43 ± 14 (11-63)
VTI, cm	19.08 ± 5.06 (12.7-29.3)
Vpeak, m/s	1.042 ± 0.234 (0.71-1.48)
CI, L/min/m^2^	3.14 ± 1.16 (1.9-6.32)
E, m/s	0.94 ± 0.27 (0.54-1.5)
DT, s	0.201 ± 0.054 (0.097-0.311)
A, m/s	0.63 ± 0.22 (0.22-1.17)
E/A	1.7 ± 1.1 (0.7-5.3)
e', cm/s	9.3 ± 3.4 (4.8-18.8)
a', cm/s	9.9 ± 3.3 (5.3-17.7)
E/e'	10.93 ± 3.98 (4.29-18.56)
s', cm/s	11.7 ± 4.2 (4.3-18.4)
**Biochemistry**	
BNP, ng/L	714 ± 882 (49-2930)
NTproBNP, ng/L	1115 ± 1234 (28-4139)
CRP, mg/L	223 ± 96 (11-394)
TnT, μg/L	0.158 ± 0.21 (0-0.71)

a', peak active (late) diastolic septal mitral annulus velocity; A, peak active (late) diastolic transmitral flow velocity; APACHE, Acute Physiology and Chronic Health Evaluation; BNP, B-type natriuretic peptide; CI, cardiac output index; CRP, C reactive protein; DT, E wave deceleration time; e', peak early diastolic septal mitral annulus velocity; E, peak early diastolic transmitral flow velocity; E/A, ratio of E to A; E/e', ratio of E to e'; EF, ejection fraction; LVEDVI, left ventricular end-diastolic volume index; LVESVI, left ventricular end-systolic volume index; NTproBNP, N-terminal proBNP; s', peak systolic septal mitral annulus velocity; SD, standard deviation; SOFA, Sequential Organ Failure Assessment; SVI, stroke volume index; TnT, troponin T; Vpeak, peak left ventricular outflow tract velocity; VTI, left ventricular outflow tract velocity time integral.

Sixteen patients (76%) were mechanically ventilated at the time of the initial assessment. The requirement for mechanical ventilation did not distinguish survivors from non-survivors (*P *= 0.15). Of the mechanically ventilated patients, positive end-expiratory pressure (PEEP) requirements were not different between survivors and non-survivors (7.05 ± 3 cmH_2_O vs 7.9 ± 5.1 cmH_2_O, respectively; *P *= 0.67).

Eleven patients (52%) were in normal sinus rhythm and two (9.5%) were paced. Noradrenaline infusion was running at the time of initial assessment in seventeen patients (81%; Mean ± SD infusion rate 0.124 ± 0.12 micrograms/kg/min). In addition to noradrenaline, one patient was receiving adrenaline and one was receiving dopamine. Mean ± SD fluid balance on the day of study was 1780 ± 1848 mL (range: -1734 to 5320).

The diagnosis of cardiac disease (Table 1) was based on previous history (non-acute) in seven out of nine patients. Of the remaining patients, one developed sepsis secondary to wound infection eight days following aortic root and valve replacement (no significant coronary artery disease; survived to hospital discharge), whereas the other developed pneumonia sixteen days following emergency coronary artery bypass grafting for acute myocardial infarction (non-survivor). No patients had a previous history of heart failure. Fourteen patients had been receiving treatment for hypertension prior to the development of septic shock. Six patients had been previously diagnosed with diabetes mellitus (n = 5; all type 2) or glucose intolerance prior to the development of septic shock.

### Echocardiography

Systolic dysfunction (EF < 55%) was evident in 14 patients (67%). Transthoracic measurement of e' and E/e' was feasible in 20 of 21 patients. Fusion of E and A waves was observed in four examinations (19%). Fusion of e'and a' waves was observed in three (15%). At initial assessment, e' was less than 9.6 cm/s in 11 (55%) patients. At this time, E/e' was more than 15 in three (15%), 8 to 15 in thirteen (65%) and less than 8 in four (20%) patients. TDI variables (including e', a', s' and E/e') were not significantly different between ventilated and non-ventilated patients. Diastolic function was graded as normal in nine (43%), impaired relaxation in three (14%), pseudonormal in seven (33%) and restrictive in two patients (10%). Thus, diastolic dysfunction was present in 57% of patients (n = 12).

### Biochemistry

BNP was elevated in fifteen patients (71%), NTproBNP in six (28%) and TnT in fourteen (67%).

### Hospital outcome

**Figure 1:**
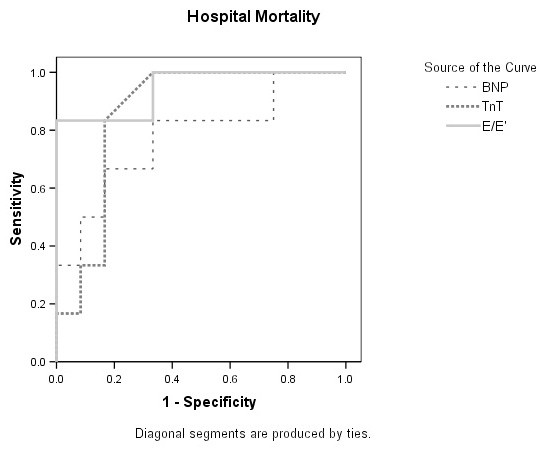
**Receiver operating characteristic curve comparing E/e' with BNP, and TnT as discriminators of hospital mortality**. BNP, B-type natriuretic peptide; E/e', ratio of peak early diastolic transmitral flow velocity to peak early diastolic septal mitral annulus; TnT, Troponin T.

Significant differences were observed between hospital survivors and non-survivors (Table 3) with respect to E/e' (survivor 9.05 ± 2.75, non-survivor 15.32 ± 2.74; *P *= 0.0002), e' (survivor 10.4 ± 3.4 cm/s, non-survivor 6.8 ± 1.9 cm/s; *P *= 0.025) and s' (survivor 13 ± 3.7 cm/s, non-survivor 8.6 ± 4.1 cm/s; *P *= 0.03). The area under the ROC curve (c statistic) for each of these variables was 0.94 for E/e', 0.86 for e'and 0.83 for s'. An E/e' threshold value of 14.5 offered sensitivity of 100% and specificity of 83% (Figure 1). The c statistic of 0.86 for TnT, 0.78 for BNP and 0.67 for NTproBNP. No difference in LVEF (systolic function) was observed (survivor 43 ± 15%, non-survivor 43 ± 14%; *P *= 0.91).

**Table 3:** Comparison of hospital survivors and nonsurvivors

Variable	Survivors (Mean ± SD)	Non-survivors (Mean ± SD)	P*	Cox Regression§
**Baseline characteristics**				
n (%)	15 (71%)	6 (29%)		
Gender, M:F	8:7	5:1	0.2 ‡	.3
Age, years	64 ± 16	67 ± 20	0.73	0.66
Height, cm	166.5 ± 8	169 ± 6.7	0.56	0.56
Weight, kg	79.1 ± 20.3	81.5 ± 14.4	0.79	0.95
BSA, m^2^	1.86 ± 0.27	1.93 ± 0.2	0.56	0.74
Cardiac disease, n (%)	5 (24%)	4 (19%)	0.18 ‡	0.17
APACHE III score (Day 1 ICU)	78.5 ± 24.9	84 ± 22.7	0.65	0.65
SOFA score (Day 1 ICU)	10.3 ± 2.6	12.3 ± 2.7	0.15	0.19
**Study day**				
Time from onset of septic shock, days	2 ± 0.8	1.7 ± 0.5	0.34	0.3
APACHE III score	80.8 ± 33.2	88.2 ± 19.1	0.62	0.77
SOFA score	10.6 ± 3.6	13.3 ± 3.2	0.14	0.23
Mechanical ventilation, n (%)	10 (48%)	6 (29%)	0.15 ‡	0.38
**Clinical monitoring**				
HR, beats/min	87 ± 15	85 ± 10	0.77	0.7
SBP, mmHg	115 ± 16	109 ± 14	0.39	0.32
DBP, mmHg	54 ± 7	48 ± 9	0.21	0.16
MAP, mmHg	72 ± 8	68 ± 10	0.38	0.3
CVP, mmHg	13.8 ± 3	14.5 ± 6	0.72	0.63
**Fluid and vasopressor management**				
Fluid balance, mL	1375 ± 1679	2792 ± 2009	0.11	0.08
Noradrenaline dose, μg/kg/min	0.115 ± 0.123	0.147 ± 0.111	0.58	0.47
**Echocardiography**				
LA area, cm^2^	22.49 ± 5.1	24.28 ± 4.45	0.46	0.35
LVEDVI, mL/m^2^	61.2 ± 19.6	83 ± 26.6	0.08	0.14
LVESVI, mL/m^2^	36 ± 20	43 ± 12	0.52	0.66
SVI, mL/m^2^	25.1 ± 10.3	30.2 ± 22.8	0.62 †	0.62
EF, %	43 ± 15	43 ± 14	0.91	0.84
OTD, cm	2.054 ± 0.243	2.23 ± 0.17	0.13	0.17
VTI	19.63 ± 4.54	17.42 ± 6.71	0.41	0.4
Vpeak	1.084 ± 0.203	0.914 ± 0.297	0.16	0.1
CI, L/min/m^2^	3.13 ± 0.96	3.18 ± 1.76	0.93	0.98
E, m/s	0.89 ± 0.24	1.04 ± 0.33	0.27	0.24
DT, s	0.215 ± 0.055	0.168 ± 0.032	0.07	0.07
E A fusion, n (%)	2 (9.5%)	2 (9.5%)	0.32 ‡	0.4
A, m/s	0.6 ± 0.18	0.73 ± 0.33	0.32	0.3
E/A	1.7 ± 1.2	1.5 ± 0.6	0.75	0.7
e', cm/s	10.4 ± 3.4	6.8 ± 1.9	0.025	0.04
a', cm/s	9.9 ± 3.4	9.9 ± 3.2	0.99	0.96
e'a' fusion, n (%)	1 (5%)	2 (9.5%)	0.18 ‡	0.15
E/e'	9.05 ± 2.75	15.32 ± 2.74	0.0002	0.005
s', cm/s	13 ± 3.7	8.6 ± 4.1	0.03	0.048
Diastolic dysfunction, n (%)	8 (38%)	4 (19%)	0.66 ‡	0.55
**Biochemistry**				
BNP, ng/L	448 ± 607	1289 ± 1155	0.14 †	0.07 ¶
NTproBNP, ng/L	841 ± 818	1801 ± 1853	0.27 †	0.2 ¶
TnT, μg/L	0.114 ± 0.174	0.268 ± 0.251	0.12	0.03
CRP, mg/L	228 ± 85	207 ± 135	0.68	0.51

*Comparison performed using Student's T test for independent groups (equal variance assumed) unless otherwise indicated. †Equal variances not assumed (Levene's test *P *< 0.05). ‡ Fisher's exact test (2 tail). §Univariate Cox regression analysis of variables as predictors of hospital survival. ¶Variables log-transformed prior to Cox regression analysis. A, peak active (late) diastolic transmitral flow velocity; a', peak active (late) diastolic septal mitral annulus velocity; APACHE III, Acute Physiology and Chronic Health Evaluation III; BNP, B-type natriuretic peptide; BSA, body surface area; CI, cardiac output indexed to body surface area; CRP, C reactive protein; CVP, central venous pressure; DBP, diastolic blood pressure; DT, E wave deceleration time; E, peak early diastolic transmitral flow velocity; e', Peak early diastolic septal mitral annulus velocity; E/A, ratio of E to A; E/e', patio of E to e'; EF, ejection fraction; F, female; HR, heart rate; LV, left ventricle or ventricular; LVEDVI, left ventricular end-diastolic volume indexed to body surface area; LVESVI, left ventricular end-systolic volume indexed to body surface area; M, male; MAP, mean arterial pressure; NTproBNP, N-terminal proBNP; OTD, LV outflow tract diameter; s', peak systolic septal mitral annulus velocity; SBP, systolic blood pressure; SD, standard deviation; SOFA, Sequential Organ Failure Assessment score; SVI, left ventricular stroke volume indexed to body surface area; TnT, Troponin T; Vpeak, peak LV outflow tract velocity; VTI, LV outflow tract velocity time integral.

#### Prediction of hospital survival

**Figure 2:**
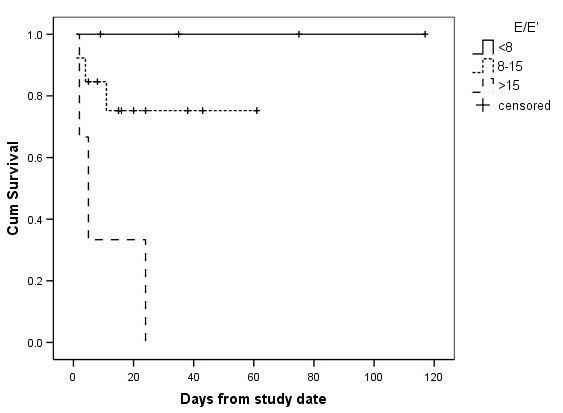
**Kaplan Meier plot of association between E/e' and hospital survival**. Cases are censored at hospital discharge. E/e', ratio of peak early diastolic transmitral flow velocity to peak early diastolic septal mitral annulus.

Univariate Cox regression analysis (Table 3) yielded significant associations between survival to hospital discharge and E/e' (*P *= 0.005), e' (*P *= 0.04), s' (*P *= 0.048) and TnT (*P *= 0.03). Adjustment for APACHE III score, history of cardiac disease, fluid balance and grade of diastolic function, revealed E/e' as an independent predictor of hospital mortality (*P *= 0.019). A Kaplan-Meier plot of the association between E/e' and survival to hospital discharge is shown in Figure 2.

### Plasma BNP concentration

From an initial model containing APACHE III score, gender, cardiac disease, fluid balance, noradrenaline dose, CRP, EF and diastolic dysfunction, the backward elimination procedure yielded a 'best' model containing gender (*P *= 0.089), APACHE III score (*P *= 0.033), fluid balance (*P *= 0.001) and diastolic dysfunction (*P *= 0.009). This final model accounted for 71.3% of variation in lnBNP concentration (adjusted R square 0.713).

## Discussion

The cardinal finding of this study is that E/e' offers independent and better prognostic prediction of hospital outcome in septic shock as compared with cardiac biomarkers (BNP, NTproBNP, TnT). We also observed that conventional measures of systolic function, such as EF and SVI (Table 3) did not discriminate between hospital survivors and non-survivors. This study also demonstrates that fluid balance and diastolic dysfunction are independent predictors of BNP concentration in septic shock patients.

### Diastolic function and tissue Doppler imaging in septic shock

We have demonstrated an association between TDI indices of diastolic function and outcome in septic shock. The significance of this important new finding is highlighted by the superiority of these variables over the measures of cardiac systolic function and cardiac biomarkers incorporated into this study. Despite demonstrating value in a range of cardiovascular diseases [42], and more recently in a study of general ICU patients by Ikonomidis and colleagues [22], the prognostic potential of TDI in septic shock *per se *has not previously been reported.

Our demonstration of an association between diastolic function and mortality in septic shock complements previous data. In a radionuclide cineangiographic study, Parker and colleagues documented that non-survivors did not demonstrate LV dilation ('preload adaptation') and therefore were unable to maintain stroke volume and cardiac output [43,44]. Also, Munt and colleagues demonstrated DT as an independent predictor of mortality in severe sepsis [45]. In addition to our TDI findings, we observed a trend toward an association between DT and hospital mortality (*P *= 0.07). Although no clear functional relation has been demonstrated, sepsis-induced diastolic dysfunction is likely to be associated with a range of histologic abnormalities such as inflammatory infiltrate, interstitial edema, apoptosis, and necrosis [46,47].

The peak early diastolic mitral annular velocity (E)', as measured by TDI, reflects LV relaxation [48,49]. Although this variable appears not to be as preload insensitive as originally proposed [49,50], it is increasingly valued as a quantitative index of LV diastolic function. This is because it does not pseudo-normalize in the same way as transmitral flow [51]. Also, the E/e' ratio has been proposed as an estimate of LV filling pressure that corrects E velocity for the influence of myocardial relaxation [33,52].

Two recent studies have utilized TDI in the evaluation of septic ICU patients. McLean and colleagues used E/e' as an estimate of LV filling pressure in their prognostic study of BNP in patients with severe sepsis and septic shock [53]. They reported E/e' to be non-significantly lower in non-survivors (survivors 14.8 ± 7.4, non-survivors 12.1 ± 4.6; *P *= 0.452). However, their study incorporated a number of patients with severe sepsis (lower severity of illness compared with the current study), and did not report fluid management, which is an important determinant of survival in sepsis [54]. Bouhemad and colleagues [55] used TDI to demonstrate isolated and reversible impairment of ventricular relaxation in septic shock patients with increased plasma troponin I concentration but associations with mortality were not assessed.

Cardiac biomarkers including BNP [23,24], NTproBNP [25] and troponin [26] have been offered as potential prognostic tools in the critically ill. Our study demonstrates the superiority of TDI over these biomarkers. This is potentially explained by the magnitude of potential confounders on plasma biomarker concentrations in the critically ill [3]. Furthermore, TDI offers more direct evaluation of myocardial function.

### B-type natriuretic peptide

In general, BNP is a peptide hormone secreted by the ventricular myocardial in response to wall stress [3]. Its principal clinical use is the diagnosis of heart failure [56]. However, elevated BNP appears to lack validity as a biomarker of myocardial dysfunction in sepsis. Potential explanations include inflammation [38], altered clearance [57], altered intrathoracic pressures/mechanical ventilation [58], vasoactive and inotropic drugs [37], fluid management [36,59], and diastolic dysfunction [40].

On the basis of previous laboratory data [36] and our own clinical research [60], we incorporated an auxiliary aim of the current study of evaluating the potential influence of fluid management on plasma BNP concentrations in septic shock. Also, the relation between diastolic function and plasma BNP concentration had not been evaluated in septic shock. We are the first to demonstrate fluid balance and diastolic dysfunction as independent predictors of plasma BNP concentration in septic shock.

### Limitations

In keeping with international guidelines for hemodynamic monitoring in shock, our unit does not routinely use pulmonary artery catheters [61], and LV filling pressure is not pursued as a therapeutic target. Although incorporation of pulmonary artery catheter data might have yielded interesting comparisons, it was unnecessary to achieve or stated aims and might have impaired the feasibility of our study. We propose that the resultant observational data forms a robust reflection of clinical practice in the context of contemporary sepsis management. Based on our findings, additional research incorporating pulmonary artery catheterization might now be justified.

We have reported TDI measurements taken at the septal mitral annulus. This technique was based on results reported by Ommen and colleagues demonstrating good prediction of LV end-diastolic pressure [33]. Although the feasibility of this approach in critical care is appealing, the mean of measurements sampled around the perimeter of the mitral valve would be less susceptible to regional wall motion abnormalities, if present [9]. The potential influence of mechanical ventilation, right ventricular function and inotropes/vasopressors upon tissue Doppler variables is unclear. Our observational study has not been designed to clarify these potential interactions but based on the current findings, further research in these areas is justified.

In clinical studies, it is challenging to standardize data collection at a fixed time from onset of sepsis. We studied patients within 72 hours of development of septic shock (admission to ICU or onset in ICU). The strength of this design is that it potentially optimizes the comparison of TDI with cardiac biomarkers, particularly BNP [23], as predictors of outcome. Due to an inability to predict the development of sepsis, we were unable to define the pre-morbid diastolic function of the study participants.

### Potential clinical significance and directions for future research

The findings of this study are of potential clinical importance. First, TDI might prove useful in risk stratification. This may help identify septic shock patients requiring more intensive therapy based upon their diastolic performance. Secondly, the association between diastolic dysfunction and mortality might offer a novel therapeutic target. Further research incorporating therapies targeted toward improved cardiac relaxation (lusitropy) must be pursued.

## Conclusions

In this preliminary study, we have found that after adjustment for severity of illness, cardiac disease, fluid management and grade of diastolic dysfunction, E/e' is an independent predictor of hospital survival in septic shock patients. In addition, E/e' offers better discrimination between hospital survivors and non-survivors than cardiac biomarkers (BNP, NTproBNP, TnT). Fluid balance and diastolic dysfunction are independent predictors of BNP concentration in septic shock.

## Key messages

• E/e' is an independent predictor of hospital survival in septic shock patients.

• E/e' offers better discrimination between hospital survivors and non-survivors than cardiac biomarkers (BNP, NTproBNP, TnT).

• Fluid balance and diastolic dysfunction are independent predictors of BNP concentration in septic shock.

## Abbreviations

A: peak active (late) diastolic transmitral flow velocity; a': peak active (late) diastolic septal mitral annulus velocity; APACHE III: Acute Physiology and Chronic Health Evaluation III; BNP: B-type natriuretic peptide; BSA: body surface area; CI: cardiac output indexed to body surface area; CRP: C reactive protein; CVP: central venous pressure; DBP: diastolic blood pressure; DT: E wave deceleration time; E: peak early diastolic transmitral flow velocity; e': Peak early diastolic septal mitral annulus velocity; E/A: ratio of E to A; E/e': patio of E to e'; EF: ejection fraction; HR: heart rate; lnBNP: multiple-linear regression analyses of BNP concentration; LV: left ventricle or ventricular; LVEDV: left ventricular end-diastolic volume; LVESV: left ventricular end-systolic volume; LVEDVI: LVEDV indexed to body surface area; LVESVI: LVESV indexed to body surface area; MAP: mean arterial pressure; NTproBNP: N-terminal proBNP; OTD: LV outflow tract diameter; PEEP: positive end expiratory pressure; ROC: receiver operating characteristic; s': peak systolic septal mitral annulus velocity; SBP: systolic blood pressure; SD: standard deviation; SOFA: Sequential Organ Failure Assessment score; SVI, left ventricular stroke volume indexed to body surface area; TDI: Tissue Doppler imaging; TnT: Troponin T; Vpeak: peak LV outflow tract velocity; VTI: LV outflow tract velocity time integral.

## Competing interests

The authors declare that they have no competing interests.

## Authors' contributions

D Sturgess conceived of the study, coordinated study design and implementation and drafted the manuscript. TM participated in study design and helped to draft the manuscript. C Joyce participated in study design and helped to draft the manuscript. C Jenkins participated in the design of the study, performed and coordinated echocardiography. MJ participated in the design of the study and provided statistical advise. PM participated in study design, provided laboratory equipment and advice regarding biochemical assays. D Stewart assisted in recruitment of participants and collection of data. BV participated in its design and helped to draft the manuscript. All authors read and approved the final manuscript.
